# Perspective on the use of methanogens in lithium recovery from brines

**DOI:** 10.3389/fmicb.2023.1233221

**Published:** 2023-08-02

**Authors:** Annalisa Abdel Azim, Arianna Vizzarro, Ruggero Bellini, Ilaria Bassani, Luisa Baudino, Candido Fabrizio Pirri, Francesca Verga, Andrea Lamberti, Barbara Menin

**Affiliations:** ^1^Centre for Sustainable Future Technologies, Fondazione Istituto Italiano di Tecnologia, Turin, Italy; ^2^Department of Environment, Land and Infrastructure Engineering, Politecnico di Torino, Turin, Italy; ^3^Department of Applied Science and Technology, Politecnico di Torino, Turin, Italy; ^4^Istituto di Biologia e Biotecnologia Agraria, Consiglio Nazionale Delle Ricerche, Milan, Italy

**Keywords:** water desalination, lithium recovery, biomethanation, critical raw material, biosorption, salinity, methanogens, brine mining

## Abstract

Methanogenic archaea stand out as multipurpose biocatalysts for different applications in wide-ranging industrial sectors due to their crucial role in the methane (CH_4_) cycle and ubiquity in natural environments. The increasing demand for raw materials required by the manufacturing sector (i.e., metals-, concrete-, chemicals-, plastic- and lubricants-based industries) represents a milestone for the global economy and one of the main sources of CO_2_ emissions. Recovery of critical raw materials (CRMs) from byproducts generated along their supply chain, rather than massive mining operations for mineral extraction and metal smelting, represents a sustainable choice. Demand for lithium (Li), included among CRMs in 2023, grew by 17.1% in the last decades, mostly due to its application in rechargeable lithium-ion batteries. In addition to mineral deposits, the natural resources of Li comprise water, ranging from low Li concentrations (seawater and freshwater) to higher ones (salt lakes and artificial brines). Brines from water desalination can be high in Li content which can be recovered. However, biological brine treatment is not a popular methodology. The methanogenic community has already demonstrated its ability to recover several CRMs which are not essential to their metabolism. Here, we attempt to interconnect the well-established biomethanation process with Li recovery from brines, by analyzing the methanogenic species which may be suitable to grow in brine-like environments and the corresponding mechanism of recovery. Moreover, key factors which should be considered to establish the techno-economic feasibility of this process are here discussed.

## 1. Introduction

The wide diversity and distribution of methanogens, unicellular obligate anaerobes from the Archaea domain, make them suitable for multiple biotechnological applications beyond high-energy fuel production, i.e., methane CH_4_ (Pfeifer et al., 2021; Bellini et al., 2022; Carr and Buan, 2022; Contreras et al., 2022; Lyu et al., 2022). Biomining and biohydrometallurgy exploit microorganisms for metal extraction and recovery from different resources such as mineral rocks, mine waste, electric and electronic waste (e-waste), new- and old-scrap metals generated during the device manufacturing and at end-of-life, respectively (Kaksonen et al., 2020; Magoda and Mekuto, 2022; Abdel Azim et al., 2023). Methanogens, in the form of consortia, have already demonstrated the ability to recover platinum group metals (PGMs) such as platinum (Pt) and palladium (Pd) (Pat-Espadas et al., 2016; Simon-Pascual et al., 2018). As a single culture, the hydrogenotrophic methanogen *Methanobacterium bryatii* BKYH was found to be able of chelating copper (Cu^2+^) from Cu-rich mineral deposits (Kim et al., 1995), while *Methanothermobacter thermoautotrophicus* could recover vanadium (V^4+^), chromium (Cr^3+^) and cobalt (Co^2+^) via bioreduction, an immobilization process which changes the oxidation state of dissolved metals by donating electrons (Zhang et al., 2014; Singh et al., 2015a,b). In this context, biobased processes are of considerable interest, being economically convenient and environmentally sustainable compared to the common techniques (Baniasadi et al., 2019). Indeed, material production typically relies on energy-consuming practices like mineral mining, processing, and refining (Intergovernmental Panel on Climate Change (IPCC), 2023). The ever-growing need for raw materials in the manufacturing industry is driving the exploration and development of alternative sources and technologies. However, the replacement of fossil-based technologies by renewable energy sources (RES) and the drive for electrification implies the exploitation of more raw materials (Zhang et al., 2023). Among raw materials, several are listed as critical (CRMs) due to their increasing demand but limited availability (Mosley, 2022; U.S. Geological Survey, 2022). Lithium (Li^+^), the demand of which is projected to grow by 32% within 2030 (Andreas et al., 2022), has been recently included among CRMs and in the strategic raw material (SRMs) list (European Commission, 2023). Li is intensively employed in single-discharge- and rechargeable-batteries construction (74%), (U.S. Geological Survey, 2022), used in electronic devices, electric (EVs) and hybrid vehicles, and smart grid factories. The demand for LIBs led the global Li production to grow from 82,500 tons in 2020 to almost 100,000 tons in 2021 along with a significant price increase of Li (as Li_2_CO_3_). However, beyond batteries, there are other well-settled applications of lithium such as ceramics and glass manufacturing, aluminum alloys for aerospace applications, as fuel in nuclear reactors (U.S. Geological Survey, 2022). Overall, the extraction procedure represents the main shortcoming in the Li supply chain in terms of energy and time demands, in addition to the use of strong reagents which makes this methodology poorly sustainable (Gruber et al., 2011; Meng et al., 2021). Total lithium resources globally account for 89 million tons (U.S. Geological Survey, 2022). Lithium only exists as salts or minerals (i.e., lithium carbonate, lithium chloride, spodumene, lepidolite, and petalite) due to its high reactivity. Hence, it can be found in hard rock ores and sedimentary rocks or water resources, including seawater and brines (Flexer et al., 2018; Baudino et al., 2022; Khalil et al., 2022; Barbosa et al., 2023). Natural brines, classified as geothermal, oilfield, and continental, are typically characterized by high salinity values with a mineral salt concentration range of 2.9–5.6 M (Flexer et al., 2018). Besides chloride Cl^−^, anions in brine include carbonates CO_3_^2−^, sulfates SO_2_^2−^ and borates BO_3_^3−^ (Talens Peiró et al., 2013). The cationic fraction is mostly represented by sodium Na^+^, potassium K^+^, magnesium Mg^2+^, and calcium Ca^2+^ in addition to less abundant elements like Li^+^ (Flexer et al., 2018), rubidium (Rb^+^) and gallium (Ga^3+^) (del Villar et al., 2023). Li content in many brines is several hundred mgL^-1^ and few brines contain more than 1 gL^−1^ of Li (Kamienski et al., 2004). Dry lakes and salt aquifers (i.e., continental brines) hold the highest concentration of Li^+^, ranging between 20 and 1,500 mgL^−1^ (Barbosa et al., 2023). The concentration of Li in marine basins such as the Atlantic Ocean and the Dead Sea is 220 μgL^−1^ and 21 mgL^−1^_,_ respectively (Barbosa et al., 2023). The *Lithium Triangle* in the Andean region among Chile, Bolivia, and Argentina accounts for up to 80% of the global lithium brine resources: among valuable commercial brines the Atacama salar in Chile has the highest lithium content besides bohrium and potassium (An et al., 2012; Ogawa et al., 2014). The current production capacity in the above-mentioned area is detained by two societies corresponding to 48,000 Li_2_CO_3_/6,000 LiCl and 27,000 Li_2_CO_3_/4500 LiCl tons per year, respectively (Flexer et al., 2018). Concentrated brines (NaCl >0.8 M), intended as the by-product of the water desalination process to produce clean water, also present massive concentrations of valuable minerals (five times the input seawater) in comparison to other brine sources (Khalil et al., 2022; Prasad, 2023). The number of elements in rejected brines varies based on the origin of the processed water: Li concentration in the Mediterranean Sea is higher than in the Atlantic Ocean but still lower than in underground brackish sources, i.e., formation waters or deep saline aquifers (del Villar et al., 2023). Hence, depending on the treated rejected water, the corresponding economic potential varies with the elemental composition (del Villar et al., 2023). The current brine production is 141.5 million m^3^ day^−1^ worldwide, 70.3 % of which is concentrated in the Middle East (Saudi Arabia, United Arab Emirates, and Kuwait) and North Africa regions. The brine is disposed directly into the ocean taking advantage of the proximity of the desalination plants to the coast, despite the related environmental risks and the volume of generated brine exceeding the volume of produced desalinated water by up to 50% (Jones et al., 2019). Although the cost estimation of Li extraction from rocks is nearly twice that of Li from brines, mineral mining is still the prevalent technology due to the limited offer of brines (Flexer et al., 2018; Meng et al., 2021). Among the existing studies on the recovery of Li as well as of other critical metals from secondary sources, bacteria (Işıldar et al., 2019; Naseri et al., 2019; Moazzam et al., 2021) and fungi (Amiri et al., 2012; Horeh et al., 2016; Bahaloo-Horeh and Mousavi, 2017) are the most represented microorganisms, while almost no data are available on the application of methanogens. Brines valorization is quite unpopular among the studies involving biological processes, though biosorption technologies are already widely applied for the treatment of other industrial wastewaters contaminated by heavy metals. Fungi, Algae and Bacteria have been broadly exploited as biosorbents (Dayana et al., 2013; Kanamarlapudi et al., 2018; Elgarahy et al., 2021; Kurniawan et al., 2023; Paper et al., 2023; Tripathi et al., 2023) compared to the Archaea whose utilization is less common (Calderón et al., 2013; Vítězová et al., 2020). Among the few studies reporting biobased treatment of brines, that of Mainka et al. investigated the use of halophilic bacteria for the degradation of organic compounds in waste brines with the goal to obtain a high-quality brine to be used as raw material (Mainka et al., 2022). The work by McAdam and Judd (2008) on the application of biological removal of anionic pollutants from concentrated waste brine on ion-exchange membranes for clean water generation should also be mentioned. In this panorama, the authors aim to open a discussion on the application of *ad hoc* methanogenic consortia for Li-brines treatment as a complementary or alternative strategy to other methodologies for industrial brine valorization. Possible mechanisms of Li recovery carried out by methanogens and the possibility to pair them with biomethanation is herein examined.

## 2. Physiology of methanogens living in briny water

Microbial diversity is very high in hypersaline environments, with the salinity gradient being an important factor for microbial community composition and species diversity (McGenity and Sorokin, 2019). Redox potential and stable anaerobic conditions are key enablers for methanogenesis occurrence. Moreover, methanogens and sulfate-reducing bacteria (SRB; Barton and Fauque, 2022) are historically in competition for common electron donors such as H_2_, formate, and acetate, in the sulfate-methane transition zone (SMTZ), hence methanogenesis also depends on sulfate concentration. SRB becomes predominant when the level of sulfate is sufficiently high to be the final electron acceptor of the above-mentioned substrates. Conversely, methanogenesis is an important process in marine and hypersaline environments, like in deeper sediments poor in sulfates (Wilms et al., 2007) in highly hydrogen-productive areas (Hoehler et al., 2001 Buckley et al., 2008). Apart from *Halobacteria* class, methanogenic archaea living at concentration of NaCl >0.2 M have been identified as halophiles (Supplementary Table S1). Most of them belong to the *Methanosarcinaceae* family including *Methanosarcina*, *Methanohalophilus*, *Methanohalobium*, and *Methanosalsum* genera.

Additionally, *Methanolobus oregonensis,* an alkaliphilic, methylotrophic methanogen, is classified as halotolerant rather than halophilic (Liu et al., 1990) due to its optimal growth with salinity <0.2 M (Didari et al., 2020). Methylotrophic methanogens cannot grow on hydrogen (H_2_) and carbon dioxide (CO_2_) or acetate, rather they use non-competitive molecules such as methanol, methylated amines and methylated sulfide as electron acceptors and formate or H_2_ as electron donors (Sorokin et al., 2018) to produce methane and gain energy (Oren, 1999). *Methanosalis* sp. *SBSPR1A*, a closely related taxon in the *Methanolobus* and *Methanomethylovorans* genera, is a methylotrophic methanogen tolerating up to 3.6 M of salinity and performing methanogenesis only from dimethylamine and trimethylamine (Bueno de Mesquita et al., 2021). Methylated amines, particularly trimethylamines, originate from glycine betaine fermentation (Welsh, 2000). Quaternary amines like glycine betaine and choline can be directly used as substrates in methanogenesis by some marine strains (i.e., genus *Methanococcoides*) without the need for syntrophic metabolism. However, only partial degradation of glycine betaine to dimethylglycine (DMG) has been reported in hypersaline environments (Watkins et al., 2014). A possible explanation is that these molecules also act as *compatible solutes*, i.e., substances fitting with microbial metabolism that accumulates in the cytoplasm to balance external osmotic pressure (McGenity and Sorokin, 2019). Two main strategies to achieve microbial osmoregulation and survival in hypersaline environments have been recognized: the salt-in and the salt-out mechanism. The former is typically used by Haloarchaea and involves the rise of salt concentrations in the cytoplasm, usually with potassium chloride (KCl; Bueno de Mesquita et al., 2021). The latter, typically used by bacteria, involves the production of compatible solutes thus avoiding salt secretion in the cytoplasm, as described in Halobacteriales. Other methyl-reducing methanogens have been identified as *Methanonatronarchaeum thermophilum* and *Candidatus* Methanohalarchaeum thermophilum, formerly related to the Halobacteria from neutral salt lakes and highly alkaline soda lakes (Sorokin et al., 2017, 2018). In saline environments, a hybrid methanogenic pathway, which uses C1-methylated compounds as electron acceptors and H_2_ as an electron donor (i.e., methyl-reduction route) can be predominant (Borrel et al., 2014). This is the case of the *Methanomassiliicoccus* genus typically found in insect/animal digestive tracts and performing a methyl-dependent hydrogenotrophic methanogenesis (Cozannet et al., 2021); this was detected in a smooth hypersaline microbial mat from Shark Bay (Wong et al., 2017; García-Maldonado et al., 2018). Although methanogenesis in hypersaline environments is typically ascribed to methylotrophic methanogens, recent studies have reported evidence of putative hydrogenotrophic methanogens presence (i.e., methanogens reducing CO_2_ to CH_4_ using H_2_ or formate) in hypersaline microbial mats and endoevaporite (García-Maldonado et al., 2015, 2018; Wong et al., 2017). Methanogens from *Methanobacteriales, Methanococcales, Methanopyrales* orders were identified in samples from these environments. Among hydrogenotrophic methanogens, *Methanocalculus* genera are representative of halophiles living in highly alkaline environments. *Methanocalculus halotolerans* was instead isolated from a hypersaline oil reservoir, with the ability to grow at up to 2 M of salinity (Ollivier et al., 1998). Representatives of the genus *Methanothermobacter* was enriched in the formation waters of a gas field, showing tolerance to salinity up to 1.5 M (Gray et al., 2009). Besides *Methanosarcina*, other abundant methanogenic communities found in anaerobic treatment plants of diary wastewaters characterized by elevated salt concentrations (Vítězová et al., 2020) correspond to the hydrogenotrophic *Methanocorpusculum*, *Methanobrevibacter*, *Methanobacterium* and *Methanoculleus* genera (Zeb et al., 2019).

## 3. S-layer and EPS mediated metal cation removal via biosorption mechanism

Taxa belonging to the archaeal kingdom are characterized by a heterogenic organization and composition of the cell membrane, although they all have in common the lack of peptidoglycan (König et al., 2014) and a lipid belayer consisting of C5-isoprenoid units linked to glycerol via ether bonds (Klingl, 2014). Almost all archaea own a protein surface layer known as the S-layer with different lattice structures. Among halophilic archaea, methanogens share the same S-layer configuration (i.e., hexagonal lattice type). Besides allowing access to nutrients, the S-layer has a cell-protective and stabilizing role in environments with extreme salinity, temperature, and pH (Rodrigues-Oliveira et al., 2017). Studies conducted on a modelled S-layer structure belonging to *Methanosarcina acetivorans*, demonstrated the role of the S-layer as a charge and size barrier preventing the access of specific molecules (Arbing et al., 2012). Selectivity for specific-target metals is a desirable quality in metal-rich waste separation and recovery technologies (Echavarri-Bravo et al., 2022). Among the methanogens, the hyperthermophilic strain *Methanocaldococcus jannaschii* has been reported to selectively adsorb dissolved Fe^3+^, Ca^2+^, Zn^2+^, Cu^2+^, and Pb^2+^ metal cations (Orange et al., 2011) due to the presence of negatively charged functional groups on the cell membrane.

In a metal-rich environment, extracellular polymeric substance (EPS) production is part of a stress-response mechanism to support the cell in reducing the metal ions availability, by chelation and sequestration as an ion exchange matrix. The EPS matrix behaves like a gel-like grid that keeps microbial cells together, supporting biofilms' adhesion on surfaces and protecting the cells from extreme environments (van Wolferen et al., 2018; Li et al., 2022; Wang et al., 2022). Carboxyl, hydroxyl, sulfate, phosphoryl, and amino groups of protein in EPS are responsible for metals biosorption (Torres, 2020). For instance, dark deposits of Pb^2+^ ions found around *Methanocaldococcus jannaschii* cells suggested a mechanism of particle fixation by the EPS (Orange et al., 2011). The study by Kurniawan and Yamamoto gave us fundamental information about the biosorption power of a natural biofilm matrix isolated from a Japanese lake: Li^+^ biosorption is a physicochemical process mainly driven by the electrostatic interaction between ion species and the negatively charged sites of the proteins in the biofilm (Kurniawan and Yamamoto, 2015). Moreover, the adsorption of Li^+^ corresponded to the parallel desorption of other cations (i.e., Na^+^, Mg^2+^, Ca^2+^ and K^+^) via an ion exchange mechanism. The biosorption process observed in this study was fast (1 min) and more performing (85 μmol g^−1^ of dry biofilm) than strong and weak cation exchange resins (18 and 33 μmol g^−1^, respectively). Concerning the use of active biomass, both bacteria and fungi showed a superior uptake capacity in the magnitude of tens and hundreds mg g^−1^ of dissolved metals (Srinath et al., 2002; Iram et al., 2015). As an example of industrial application, Artola and colleagues designed and operated a biosorption pilot plant for Cu^2+^ removal from municipal water treatment plant using anaerobic sludge as biosorbent (Artola et al., 2001). The highest uptake capacity was 75 mg of metal g^−1^ of total solids in the sludge. Pagliaccia and coworkers investigated the efficiency of EPS in native biomass from annamox granular sludge as biosorbent of heavy metals in synthetic wastewaters (Pagliaccia et al., 2022). A recent study on a methanogenic consortium revealed the relationship of EPS with the release of soluble biogenic products and with metal solubility in the presence of elevated cobalt (Co^2+^) and nickel (Ni^2+^) concentrations, as in waste streams of metallurgical and LIBs industry (Hasani Zadeh et al., 2022). Hydroxyl and carboxyl terminals of proteins in EPS are the main ones responsible for the metal-cations biosorption mechanism (Fomina and Gadd, 2014; Kurniawan and Yamamoto, 2015; Liu et al., 2015) because cationic species are predominant among metals in aqueous solutions. This means that pH around 7-8 is the most suitable range for ensuring a negative charge on the protein terminals that bind dissolved metals (Torres, 2020). The concentration of dispersed metals is dependent on EPS protein content whose variation is caused by metal-induced stress, e.g., concentrations of essential or non-essential metals that exceed the cells requirement. For instance, the activity of a methanogenic consortium in an anaerobic granular sludge was compromised by both Ni and Co as reported by Hasani Zadeh and colleagues. Moreover, the presence of a Ni-protein complex proved the selective metal-binding based on the ligand affinity in metalloproteins (Hasani Zadeh et al., 2022).

EPS can also host biotransformation process as it is for *Methanococcus maripaludis OS7* producing an extracellular Ni-Fe hydrogenase that oxidizes iron of carbon steel oil and gas pipelines. The hydrogenase has the function of producing hydrogen and triggering the microbially influenced corrosion phenomenon (Lahme et al., 2021). Although archaeal EPS do not have a relevant role at the industrial level yet, its importance is progressively growing. EPS production in archaea (i.e., *Halobacterium mediterranei*) is currently estimated to be at TRL 2, based on (Pfeifer et al., 2021). There are different strategies to improve the microbial recovery mechanisms with the purpose of transferring this technology to the industrial scale such as surface-culture immobilization (fixation, entrapment, and chemicals cross-linking) and optimal conditions for process implementation (e.g., temperature, pH, initial dissolved metals, biosorbent concentration, i.e., biomass or EPS concentrations, biosorbent/metal contact time; Fomina and Gadd, 2014). As a successful example is worth to mention the study by Manasi and colleagues using a halophilic bacterium *Halomonas* BVR 1 in combination with reduced graphene oxide to remove Cd, Zn and Pb from real effluent from electronic manufacturing sector: metals removal efficiency achieved 98% (Manasi et al., 2018).

In addition to the biosorption mechanism, which is not necessarily dependent on living microorganisms, metals recovery can also occur via bioaccumulation or else through metals uptake via passive or active transport trough the cell membrane, and biotransformation and bioprecipitation, which instead involve active cells (Gavrilescu, 2022; Liapun and Motola, 2023).

## 4. Conclusion and implications for future research

**Figure 1:**
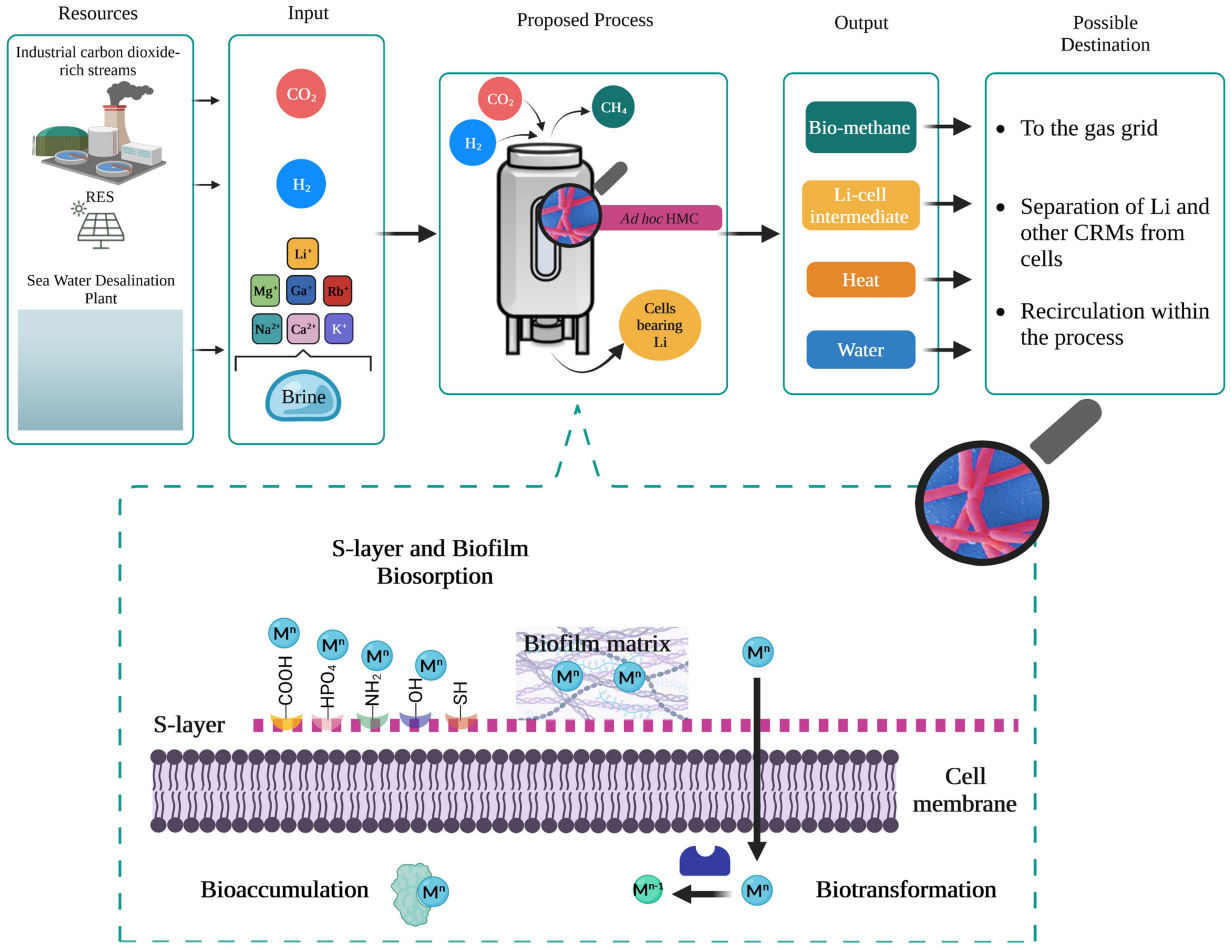
Li removal from rejected brine through biomethanation process. Sources, input, and output with possible destination are shown. The bioreactor design here is generic: biosorption at the industrial level has been investigated in stirred tank bioreactors, air lift bioreactors, fluidized bed bioreactors, and fixed bed bioreactors (Kanamarlapudi et al., 2018). Zoom in on the mechanisms enabling the recovery of metals in brine. RES, renewable energy sources; HMC, hydrogenotrophic methanogens consortia. M^n^ and M^n−1^: charge of target metals.

Brine disposal is an emerging environmental and economic issue not only for the drinking water supply chain, considering that 41% of the global population still does not have access to it, but also for the primary sector (e.g., agroforestry, zootechnic and mining) and secondary sector (e.g., metallurgy) where water is an essential resource. Therefore, desalination is expected to expand rapidly, and so brine production is associated with it. The ecological effect of direct brine discharging in surface water bodies is currently under discussion due to the related-potential physiochemical alteration and the associated threat to marine ecosystem and life. Hence, valorization of rejected brines rather than direct disposal represents the core of the future water-resources management. Extraction and recovery strategies of valuable metals from secondary sources must be expanded and implemented. Among the valuable metals lithium is particularly attractive because its demand is expected to increase enormously by 2030. Emerging technologies relying on biological approaches are very promising in terms of low cost and sustainability but require further investigation to enable their application on a large scale. Based on what is currently known, we suggest that natural-adapted consortia of methanogens could be exploited as a flexible platform for the selective recovery of Li and other critical metals from brines in a CO_2_-upcycling process (Figure 1). Thus, primary and/or secondary sectors emitting CO_2_ as a waste effluent represent a valuable source of carbon that supports the growth and productivity of methanogens. From an industrial point of view, examples of pilot and demonstration scale biomethanation plants are available in the literature with the technology being widely investigated (TRL >5). Even though biomethanation technologies appear mature and deployable, integration of Li recovery from brines would require the evaluation of some additional factors concerning resources and process operating conditions.

In particular, future research should investigate the ability of methanogenic consortia to adapt and grow on brines as substrates while carrying out methanogenesis. It is indeed important to test the resistance to salinity stress and elements which are not essential to their metabolism (e.g., Li, Sr, F). In order to maintain stable biomethane generation provided levels of salinity and thus Li should be kept under the threshold of inhibition, thus dilution of brine might be required along with culture preadaptation steps. Moreover, the optimal conditions to favor selectivity toward specific metals should be explored with the view of scaling-up the bio-recovery process. When using mixed microbial communities defining the organisms actively contributing to metal recovery and their affinity towards the removal of different brine components should be considered to eventually develop a functional synthetic consortium. In this regard, the location of origin and the type of water resource should be considered as important factors affecting the bioprocess and its profitability due to the different elemental composition. Considerable attention should be paid also to the recovery mechanism (biosorption, bioaccumulation, and biotransformation) carried out by the involved methanogenic species (Figure 1) in order to define the best implementation strategies for microbial recovery optimization (e.g., cell-immobilization). This aspect is also crucial to evaluate and deploy technically and economically feasible downstream procedures for Li desorption from cells and possibly the regeneration of the biosorbent, i.e., active methanogens, which is required by the biomethanation process. Given the lack of knowledge on the biological recovery of CRMs from rejected brine even at the laboratory scale, a techno-economic assessment of the research and development target must be still explored in order to reveal the potential benefit of this process. However, the use of renewable sources, the CO_2_ mitigation and utilization, and eventually, the heat and water generated along with the production of CH_4_ and reused within the process itself, should be an added value contributing to the process feasibility.

## Data availability statement

The original contributions presented in the study are included in the article/Supplementary material, further inquiries can be directed to the corresponding author.

## Author contributions

AA: conceptualization and writing—original draft preparation. AA, AV, RB, and IB: investigation and visualization. BM: supervision. FP: funding acquisition. AA, AV, RB, IB, LB, FP, FV, AL, and BM: writing—review and editing. All authors contributed to the article and approved the submitted version.

## Conflict of interest

The authors declare that the research was conducted in the absence of any commercial or financial relationships that could be construed as a potential conflict of interest.

## Publisher’s note

All claims expressed in this article are solely those of the authors and do not necessarily represent those of their affiliated organizations, or those of the publisher, the editors and the reviewers. Any product that may be evaluated in this article, or claim that may be made by its manufacturer, is not guaranteed or endorsed by the publisher.
